# Research on the method of designing cap lens using ant colony algorithm

**DOI:** 10.1038/s41598-021-95518-1

**Published:** 2021-08-05

**Authors:** Wenlin Liu, Hua Qin, Zhiwei Lv, Yanze Feng, Yuetong Shi, Zixiang Liu, Chenghao Bai

**Affiliations:** School of Physics and Optoelectronics Engineering, Shandong University of Technology, Zibo, Shandong China

**Keywords:** Applied optics, Lasers, LEDs and light sources, Optical physics, Optical techniques

## Abstract

It is necessary to select the appropriate parameters defining a aspheric lens for coupling the light from a laser diode into the optical fiber by cap aspheric lenses. In this paper, the ant colony optimization algorithm is applied to the optimization of structural parameters of the cap aspheric lens, and the merit function defining the optimization problem and detailed design steps are given. A cap aspheric lens with center thickness of 1.1019 mm and effective focal length of 1.10331 mm is designed using a self-made MATLAB program of ant colony optimization algorithm, which can couple the light emitting from a laser diode into a single mode fiber with a diameter of 9 um, the light-emitting surface of the LD is 3 µm × 2 µm, and beam-divergence angle in the X and Y directions are ± 35° and ± 23.58°, respectively. The theoretical coupling efficiency is 89.8%, and the experiment shows that the maximum coupling efficiency and average coupling efficiency are 88.63% and 79.39%, respectively. Design and experimental results prove that the design method in this paper is feasible and effective.

## Introduction

The transistor outline (TO) package comprises a header and a cap^1^. From a optoelectronic perspective*,* the caps can fulfill two primary functions. Firstly, they can provide durable and reliable protection for optical emitting, transmitting and receiving elements. Secondly, as an optical interface, the caps also ensure the effective transmission of optical signals between a laser diode and a fiber receiving end. The rays emitted by the laser diode are focused into the fiber end face under the action of a cap lens, by adjusting the optimal position of the lens and the optical fiber, the received light intensity at the fiber end face is maximized. Therefore, the optical properties of the window or the lens installed in the cap must meet very high requirements. The cap lens that has been used for optical fiber coupling include a molten glass ball^2^, a single ball lens^3,4^, a multi-lens system^5^, an aspheric lens^6–8^, etc.

The molten glass ball scheme is that glass cubes placed on the metal shell are melted at high temperature and collapsed into the torispherical shapes under the action of gravity and surface tension^2^. This is an early stage in the product development process, the glass balls made in this way have poor consistency and low coupling efficiency. A single ball lens belongs to a standard spherical lens. It is simple in manufacture, small in space structure, convenient in assembling and adjusting, and higher in the coupling efficiency than that of a molten glass ball. A single ball lens has become one of the most commonly used coupling lenses for SLD-SMF. However, a single ball lens itself cannot correct the spherical aberration, and the focal spot diameter is often larger than the core diameter of a fiber to be coupled. Theoretically, the maximum coupling efficiency from a laser diode (LD) to an optical fiber through a ball lens can only reach 48%^9,10^, which cannot meet the requirements of 5G communication. To obtain a high coupling efficiency with lens systems, the spherical aberration of lenses must be reduced. The multi-lens system or an aspheric lens system can effectively correct the spherical aberration, but multiple lenses increases the overall length of the lens system and make it difficult to assemble in a marrow space. A single aspheric lens can effectively eliminate spherical aberration^11^, also has the characteristics of convenient packaging as a single-lens, so a single aspheric lens coupling is widely used in optical fiber coupling systems, and becoming the mainstream of cap lenses.

Literature^7^ depicts the light field at the plane of a lens palced an optical fiber’s end face from the view of Gaussian beam theory and the ABCD propagation law, and three kinds of aspheric microlenses on fiber endfaces with different technical parameters are designed and manufactured according to these theory. By eliminating the aberrations, especially spherical aberrations, the literature^12^ presents a coupled aspheric lens system designed with the commercial optical design software OSLO. Literature^13^ designs a biconvex microlens by Zemax software to maximize the light coupling between an edge emitting laser diode (LD) and an optical fiber. Both convex surfaces have different profiles along their x-and y-axis so that the elliptical light beam from an LD can be fed into the core of a fiber. Focal spot diameter around 10 µm is achieved experimentally. Light coupling efficiency using this single biconvex microlens is 31.8% and 47.6% for single-mode and multi-mode fiber, respectively.

Current specialized CAD tools offer mostly local optimization, but only after a human has produced a draft design^14^. The ant colony algorithm belongs to a global algorithm, has strong global search ability, can perform parallel and distributed computing, and has fast convergence speed and strong adaptability^15–17^. Applying the ACA to solve complex lens system design problems might get a pretty good result without the given initial structure.

Thus, in this paper, the ant colony algorithm is introduced into the design of aspheric coupling lenses. The merit function for a coupling system is constructed by ray tracing and fiber coupling conditions based on geometrical optics, and an indirect functional connections between the merit function and the structural parameters of the aspheric lens is established through an accurate ray tracing calculation. The structural parameters is optimized to yield the best merit function by using a self-compiled MATLAB program of ant colony algorithm, so as to obtain an aspheric surface profile with the best light coupling efficiency. The coupling efficiency of the designed aspheric lens has been analysed theoretically and experimentally.

## Laser source parameters, aspheric lens and fiber coupling conditions

### Light path structure and laser parameters

**Figure 1:**
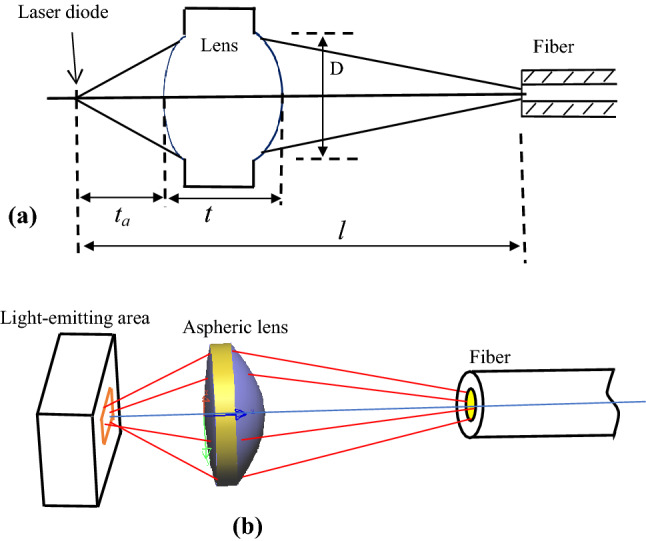
Schematic diagram of laser diode to optical fibre coupling configuration via aspheric lens. (**a**) The length parameters in coupling optical path (**b**) the three-dimensional map of coupling light path.

Figure 1a is a schematic diagram of an actual coupling lens's light path*,* and Fig. 1b is a schematic of its light path in three-dimensional space. It can be seen from Fig. 1 that light rays from a laser diode (LD) are fed into optical fibers through a single aspheric lens. The distance from a light-emitting surface to an aspheric lens is *t*_*a*_ = 0.975 mm. The center thickness of a lens is between 1 and 1.3 mm, its clear aperture is about D = 1.2 mm. The location of a receiving end of an optical fiber is estimated to be *l* = 6.23 mm away from a surface light source.

The relative position between the light-emitting surface of the semiconductor laser and the incident end of optical fiber is constant, and the characteristic of light-emitting and optical fiber are also fixed. What need to be designed is the coupling lens. An aspheric biconvex lens is adopted, and the coupling efficiency of the optical fiber can be maximized by properly selecting the aspheric surface profile.

**Figure 2:**
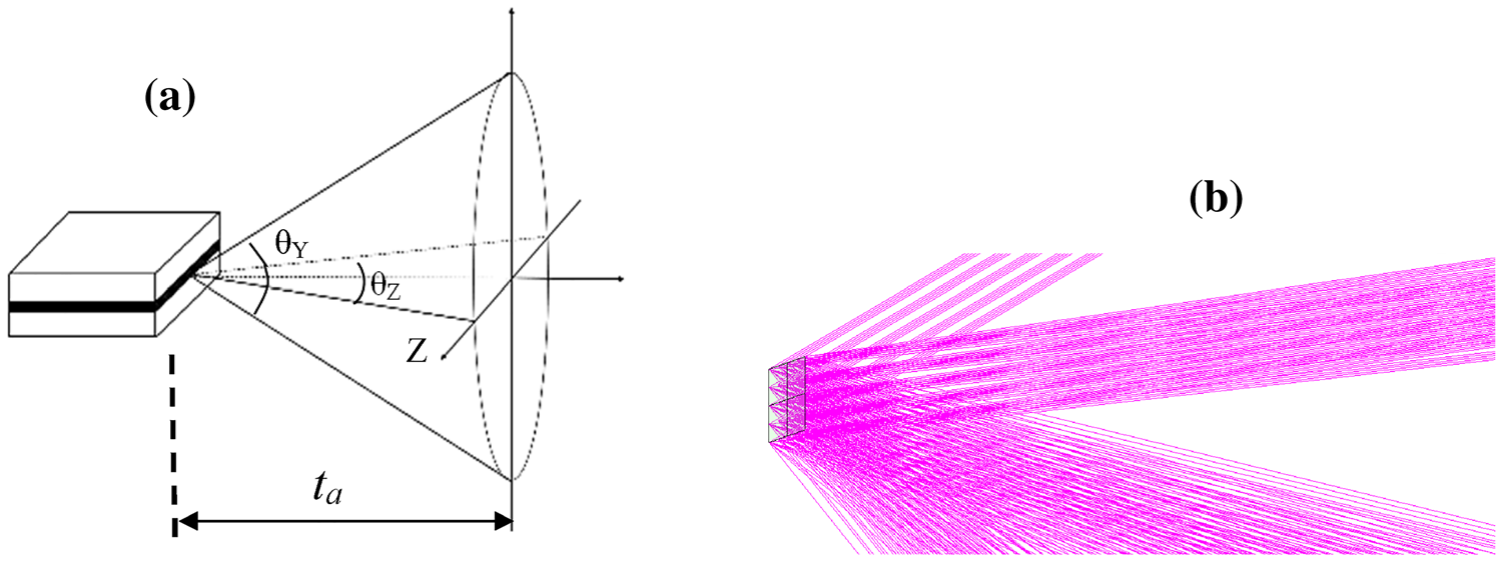
The schematic diagram of emergent light from laser diode. (**a**) The emergent beam structure diagram, (**b**) the simulation of rays from the rectangular light-emitting surface.

The parameters of light source include the size of the emission zone and the divergence angle of outgoing beam^18^. In the field of optical fiber communication technology, the emission zone of the electroluminescent chip is usually known about 3 µm × 2 µm, and the laser beam divergences are about $\frac{{\theta_{Y} }}{2} = \pm 35^\circ$ in the Y direction and $\frac{{\theta_{Z} }}{2} = \pm 23.58^\circ$ in the Z direction. The emergent light spot takes the shape of an ellipse as shown in Fig. 2a. The semi-major axis ${\varvec{a}}$ and semi-minor axis ${\varvec{b}}$ of the elliptic spot are $\user2{a = }t_{a} \tan \left( {\frac{{\theta_{Y} }}{2}} \right) = 0.975 \times tan\left( {35^\circ } \right){ = }0.68270$, ***b*** = $t_{a} \tan \left( {\frac{{\theta_{Z} }}{2}} \right) =$ 0.975 $\times$ *tan*(23.58°) = 0.42556, respectively. The equation describing an elliptic spot is written as $\frac{{y^{2} }}{{a^{2} }} + \frac{{z^{2} }}{{b^{2} }} = 1$. The entrance pupil is set as an ellipse when we design an aspheric lens.

Figure 2b presents the simulation of rays from the rectangular light-emitting surface of LD. Take a limited number of light-emitting points uniformly or according to Gaussian distribution, and each ray from any light-emitting point carries the same energy. Considering the computer’s speed, the number of light-emitting points and rays should be taken appropriately in the design of an aspheric lens.

### Aspheric lens for coupling

**Figure 3:**
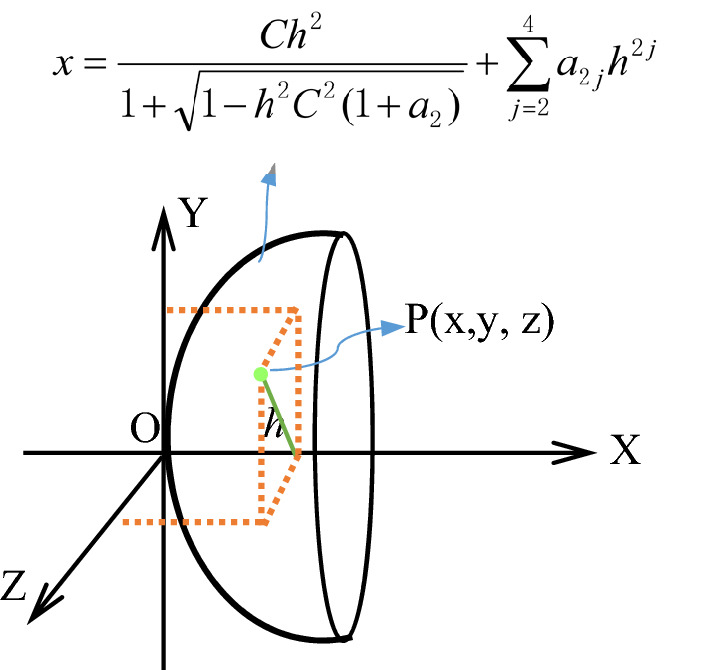
The geometric meaning of aspheric equation.

Both surfaces of a coupling lens are aspheric, and the equation to define the aspheric shape of the lens surface is as follows:

1 $$ x = \frac{{Ch^{2} }}{{1 + \sqrt {1 - h^{2} C^{2} (1 + a_{2} )} }} + a_{4} h^{4} + a_{6} h^{6} + a_{8} h^{8} $$

where $x$ is the axial value along the optical axis starting with the intersection of each aspheric surface and the optical axis, and $a_{2}$ is the quadric coefficient, *C* is the apex curvature of a aspheric surface, $h = \sqrt {y^{2} + z^{2} }$ is a vertical height from a point on an aspheric surface to the optical axis, *a*_4_, *a*_6_ and *a*_8_ is the high order coefficients of aspheric surface equation. Figure 3 gives a geometrical interpretation of Eq. (1).

The lens material is chosen to be K-VC89 glass which has excellent optical properties, good transparency at IR spectrum. The optical refraction index for K-VC89 is 1.78331 at a wavelength of 1310 nm.

### Coupling conditions for lasers into the fiber and merit functions for the iterative optimisation algorithm

The basic conditions for the laser beam to be fully coupled into a fiber are^19^

2 $$ d_{in} < d_{core} $$

3 $$ \theta_{in} < \theta_{max} = 2{\text{arcsin}}\left( {NA} \right) $$

where $d_{in}$ is the spot diameter of the laser beam on the receiving end face of the fiber,${ }d_{core}$ is the core diameter of the fiber,${ }\theta_{in}$ is the divergence angle of the laser beam and $NA$ is the numerical aperture of the fiber. Namely, the spot diameter of incident beam should be smaller than the fiber core diameter, and the incident beam divergence angle should be smaller than the acceptance angle $\theta_{max}$ of an optical fiber.

In this paper, the optical fiber core diameter is *d*_*core*_ = 9 µm, and the numerical aperture of optical fiber is NA = 0.16, that is $\frac{{\theta_{max} }}{2} = 9.2^\circ$.

Assuming that each light-emitting point on a chip emits N rays. These rays pass through an aspheric lens and intersect on the plane at the receiving end of the optical fiber, and the intersection points form a spot diagram. The mean of the distances from these intersections to the center of the fiber is defined as the size D of a spot diagram, as one of merit functions.

4 $$ D = \frac{1}{N}\mathop {\mathop \sum \limits^{N} }\limits_{k = 1} \sqrt {y_{k}^{{{\prime }2}} + z_{k}^{{{\prime }2}} } $$

**Figure 4:**
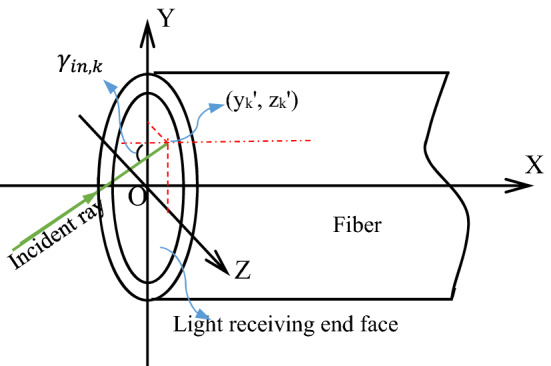
Intersection coordinates and included angle of the *k*th ray incident on the light receiving end face.

Among them, $y_{k}^{{\prime }}$, $z_{k}^{{\prime }} \left( {k \le N} \right)$ is the intersection coordinates of the ***k***th ray and the plane at the receiving end of the optical fiber, which can be obtained by ray tracing^20^. In order to make the half divergence angle of the beam incident on the fiber end face be less than 9.2°, add an angle value in radians to Eq. (4) and give appropriate weight q, then Eq. (4) becomes Eq. (5)

5 $$ MF = \frac{1}{N} \mathop {\mathop \sum \limits^{N} }\limits_{k = 1} \left( {\sqrt {y_{k}^{{{\prime }2}} + z_{k}^{{{\prime }2}} } + q\gamma_{in,k} } \right) $$

where $\gamma_{in,k}$ is the included angle of the *k*th ray incident on the fiber end face and the fiber axis, which is also calculated by ray tracing^20^, and q is a weighting factor. MF is the merit function of a coupling aspheric lens, and it is also taken as the objective function of the optimization process using the ant colony algorithm. Generally speaking, the smaller the MF value, the better the coupling aspheric lens. To illustrate, Fig. 4 shows $y_{k}^{{\prime }}$,$z_{k}^{{\prime }}$ and the induced angle $\gamma_{in,k}$.

## Method to design coupling lens using ant colony algorithm

### Constructing the solution space, namely, the ant's position vector and its search range

A single coupling lens has two faces, and the shape of each face is determined by its vertex curvature *C* and aspheric coefficient *a*_2_, *a*_4_, *a*_6_, *a*_8_. These parameters and the distance *t* between two aspheric surface’s vertices determine the complete shape of a lens. Designer’s job is to find an appropriate combination of these parameters to minimize the merit function.

It can be seen from the above analysis that the system has 11 parameters to be choosen, that is to say, there are 11 variables in the optimization process, and the ant's position vector has 11 dimensions, which can be written as follows:

6 $$ \vec{R}_{i} = \left( {R_{i1} ,R_{i2} , \ldots ,R_{i11} } \right) = \left( {C_{1} ,C_{2} ,t,a_{2,1} ,a_{4,1} ,a_{6,1} ,a_{8,1} ,a_{2,2} ,a_{4,2} ,a_{6,2} ,a_{8,2} } \right) $$

where $\vec{R}_{i}$ represents the *i*th ant’s position vector, $C_{1}$ and $C_{2}$ represent the vertex curvature of the front and back surfaces of the lens, respectively, $a_{2,1}$ and $a_{2,2}$ represent the aspheric coefficients $a_{2}$ of the first surface (front surface) and the second surface (back surface), and so on.

The ranges of the position vector can be set freely according to the actual requirements. For example, the front surface of the lens can’t be concave, so the value of $C_{1}$ should not be negative, etc. Table 1 lists the value range of each dimension of an ant’s position vector, in which $C_{1} = 1/{\text{r}}_{1}$, ${ }C_{2} = 1/{\text{r}}_{2}$.

**Table 1:** Value ranges of aspheric surface coefficients *a*_2_, *a*_4_, *a*_6_, *a*_8_ and t.

*r*_1_	*r*_2_	*t*	*a*_2,1_, *a*_2,2_	*a*_4,1_, *a*_4,2_	*a*_6,1_, *a*_6,2_	*a*_8,1_, *a*_8,2_
[0, 5]	[− 5, 0]	[1.0, 1.5]	[− 50, 50]	[− 5 × 10^–2^, 5 × 10^–2^]	[− 6 × 10^–2^, 6 × 10^–2^]	[− 6 × 10^–3^, 6 × 10^–3^]

### Initializing each ant’s position vector

Initializing the ***i***th ant’s position vector within the value range of the ant position vector as shown in Table 1:

7 $$ R_{i} \left( {0,{\text{dim}}} \right) = Rlower_{i} \left( {dim} \right) + \left[ {Rupper_{i} \left( {dim} \right) - Rlower_{i} \left( {dim} \right)} \right] \times rand $$

where ${ }R_{i} \left( {0,{\text{dim}}} \right)$ is the initial value of the *i*th ant's position vector in the dim-th dimension,${\text{rand}} \in [0,1]$ is a random number uniformly distributed in [0 ~ 1], $Rupper_{i} \left( {dim} \right)$ and $Rlower_{i} \left( {dim} \right)$ are the upper and lower bounds of the *i*th ant’s position vector in its dim-th dimension, respectively, which is listed in Table 1.

### Ant’s pheromone *τ*_*i*_ (*T*)

The ant’s position vector is actually a set of parameters defining an aspheric lens, and the merit function $MF\left( {\vec{R}_{i} } \right)$ of coupling aspheric lens can be obtained by ray tracing, which is also the objective function in optimization program. The initial value $\tau_{i} \left( 0 \right)$ of each ant’s pheromone is the merit function of the coupling lens, namely $\tau_{i} \left( 0 \right) = MF\left( {\vec{R}_{i} } \right)$, here $\vec{R}_{i}$ is the ant's initial position vector. The ants update the pheromone directly after a move from one node to an adjacent node, and the update method is as follows.

Current pheromone value = old pheromone residue + merit function at ant’s current position, old pheromone residue = (1 − $\rho$) × old pheromone. So, the pheromone $\tau_{i} T$ of the *i*th ant at its *T*th movement may be written as

8 $$ \tau_{i} T \leftarrow \left( {1 - {\uprho }} \right) \times \tau_{i} T - 1 + MF\left( {\vec{R}_{i} } \right)\quad T = 1,2,3, \ldots $$

In this paper, pheromone’s evaporation rate ρ = 0.8, Seeking maximum and minimum values can be transformed into each other by equation $\max \left[ {{\text{MF}}\left( {\vec{R}_{i} } \right)} \right] = - \min \left[ {{\text{MF}}\left( {\vec{R}_{i} } \right)} \right]$.

### Ants move around the search space to find the optimal target

Calculate the ith ant’s state transition probability Pi(*T*) at its Tth movement from existing known pheromones

9 $$ P_{i} \left( T \right) = \left[ {\tau_{i} (T - 1) - \tau_{ - best} (T - 1)} \right]/\tau_{i} (T - 1) $$

In the above equation,$\tau \_best(T{ - }1)$ is the global best-so-far pheromone value of the ant colony. When $P_{i}$(T) is greater than the transition probability constant $P_{0}$, the *i*th ant conducts a global mobile search, otherwise, local mobile search. Ants move according to the following equation

10 $$ R_{i} \left( {T,dim} \right) = \left\{ {\begin{array}{*{20}l} {R_{i} \left( {T - 1,dim} \right) + \left[ {Rupper_{i} \left( {dim} \right) - Rlower_{i} \left( {dim} \right)} \right] \times 0.1\lambda } \hfill & {P_{i} \left( {T - 1} \right) < P_{0} } \hfill \\ {R_{i} \left( {T - 1,dim} \right) + \left[ {Rupper_{i} \left( {dim} \right) - Rlower_{i} \left( {dim} \right)} \right] \times \left( {rand - 0.5} \right)} \hfill & {P_{i} \left( {T - 1} \right) \ge P_{0} } \hfill \\ \end{array} } \right. $$

$\lambda = 1/T$ is the mobility factor, which decreases with the increase of the iterative movement times *T*, $R_{i,dim,T} \in \left[ {Rlower_{i,dim} ,Rupper_{i,dim} } \right]$.

It can be seen from the transition probability rule (9) that the closer to the global best-so-far value the pheromone trails on a path at (*T* *−* 1)th iteration is, the smaller *P*_i_(*T*) is, ants tend to perform fine-tuning, that is, local search; on the contrary, the farther away from the global best-so-far value, the larger *P*_i_(*T*) is, and ants tend to search in a large range, that is the global search.

### Outputing optimal solution

The serial number of the ant with the best pheromone is output after the the maximum number of iterative movement *T*_max_, this ant’s position vector $\left( {C_{1} ,C_{2} ,t,a_{2,1} ,a_{4,1} ,a_{6,1} ,a_{8,1} ,a_{2,2} ,a_{4,2} ,a_{6,2} ,a_{8,2} } \right){ }$ is the optimal combination of aspheric parameters to be sought. Otherwise, went back to 3.3.

## The results of design and analysis

### Design results and simulation analysis

According to the description in “Method to design coupling lens using ant colony algorithm” section, the MATLAB program is compiled by ourself. Take uniformly 25 light-emitting points on a 3 µm $\times$ 2 µm rectangular luminescence surface. The entrance pupil is placed at the vertex of the front aspheric surface in the shape of an ellipse, the semi-major axis a = 0.68270, and the semi-minor axis b = 0.42556. Take 25 incidence point on the elliptic pupil, in this way, there are 625 rays to be traced for designing a lens. The Matlab program runs for about 1 h on the PC(an Intel (R)core (TM) i5-2300 CPU @ 2.8 GHz processor with 4 GB of ram), the optimal values of $\left( {C_{1} ,C_{2} ,t,a_{2,1} ,a_{4,1} ,a_{6,1} ,a_{8,1} ,a_{2,2} ,a_{4,2} ,a_{6,2} ,a_{8,2} } \right)$ are obtained as shown in Table 2, and the merit function is MF = 0.0497. It should be noted that MF values is closely related to the number of traced rays, which cannot be used for the final evaluation of design results.

**Table 2:** Lens configuration data C, *t*, *a*_2_, *a*_4_, *a*_6_ and *a*_8_ obtained by the ACA.

Surface	Curvature(*C*)	Thickness(*t*)	*a*_2_	*a*_4_	*a*_6_	*a*_8_
1	1/2.527396	1.101900	− 22.415752	− 0.030730	0.015847	0.007523
2	− 1/1.061810		− 1.195575	− 0.020387	− 0.006450	0.008327

**Figure 5:**
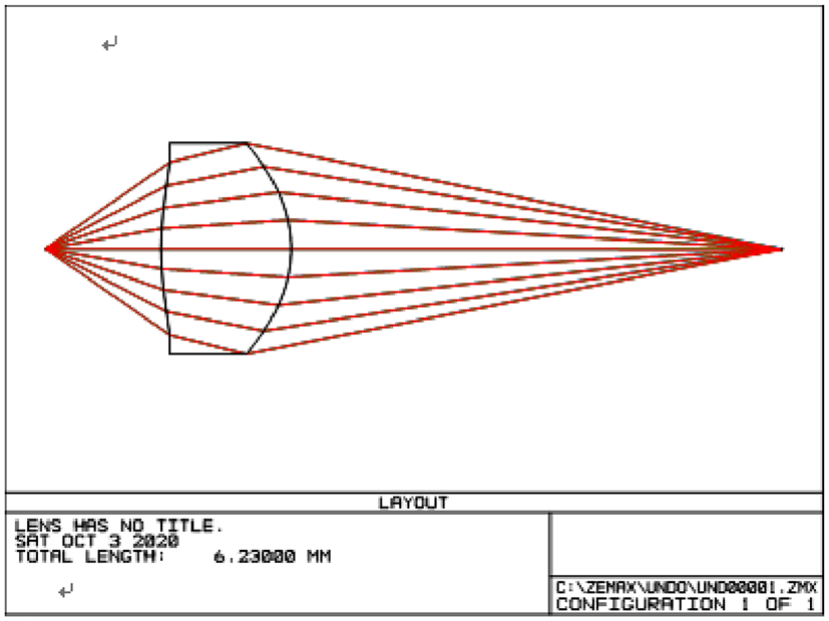
2D optical layouts plotted by Zemax for the designed coupling aspheric lens.

**Figure 6:**
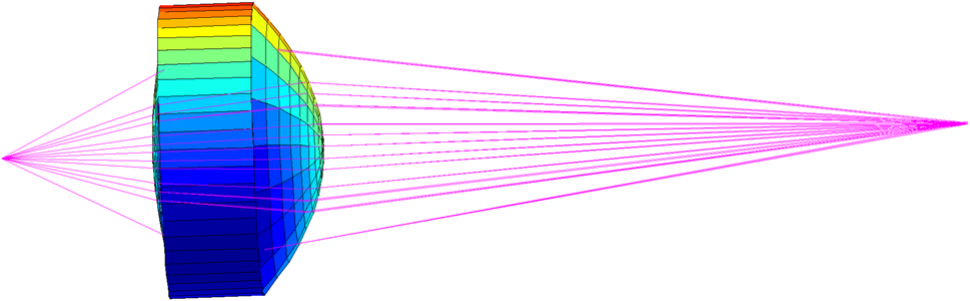
3D optical layouts plotted by plotted by the self-made MATLAB program.

Substituting these optimal parameters into the Lens Data Editor of Zemax (Zemax, version 2003), Fig. 5 shows 2D optical layouts plotted by Zemax for the designed coupling aspheric lens used in coupling caps. It can be seen from Fig. 5 that laser beams entering the entrance pupil could be focused to a very small area, and the aspheric lens has an easy-to-make shape. It can be seen from Zemax Prescription Data that the aspheric lens has the vertical axis magnification of 2.945195. Figure 6 gives the three-dimensional shape and coupling optical path of the designed lens plotted by the self-made MATLAB program (Matlab R2009a). It can also be seen from Fig. 6 that the aspheric lens designed using the method of this paper has a good focusing performance. The theoretical calculation by tracing 32,969,025 rays shows that the coupling efficiency of the designed aspheric lens can reach up to 89.8%.

**Figure 7:**
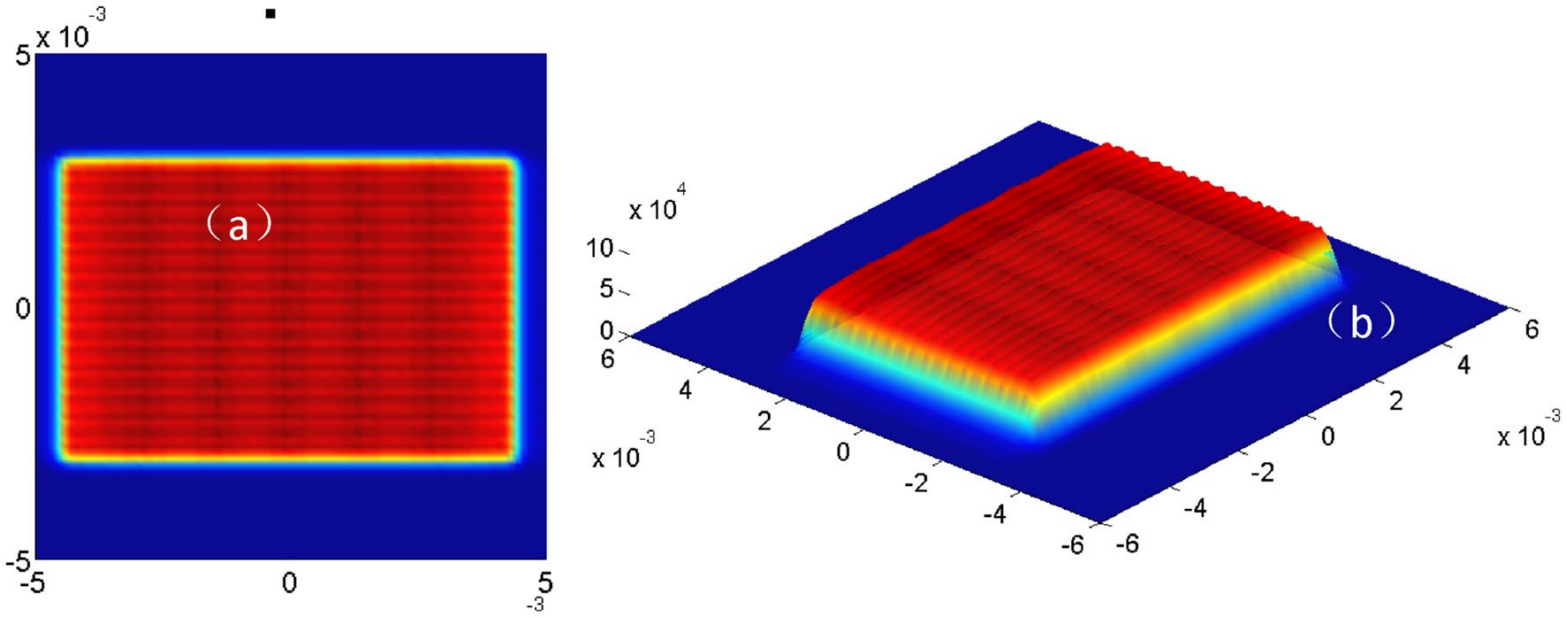
The simulation diagram of the spot shape at the receiving end face of the optical fiber, (**a**) two-dimensional spot pattern, (**b**) three-dimensional spot pattern.

Figure 7 is the simulation diagram of light spot at the receiving end face of optical fiber plotted by using the self-compiled MATLAB program tracing 32,969,025 rays. It can be seen from Fig. 7 that the light spot is the same shape as the luminance surface. The size of light spot is about 9 µm × 6 µm, which indicates most of the light is within the fiber core (*d*_*core*_ = 9 µm). The light spot shape also manifest that the coupling lens not only plays a role of coupling but also has the imaging characteristics, and the vertical axis magnification of about 3 can be calculated from this spot, which is approximately equal to the vertical axis magnification 2.945195 shown in ZEMAX. The above analyses also prove the correctness of the design results and design methods in this paper.

### Experimental analysis

**Figure 8:**
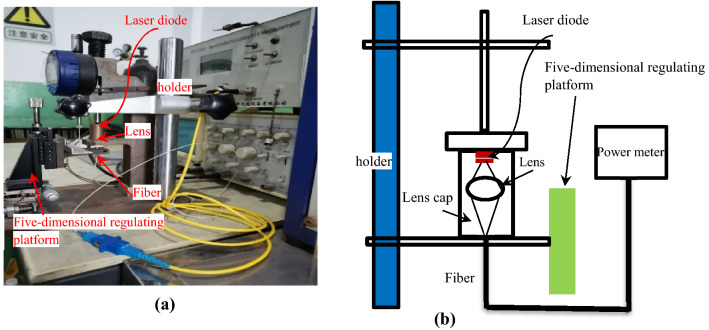
Experiment of asphetic lens coupling lights from laser diode to single mode fiber (**a**) Photo of experimental setup for measuring the optical coupling efficiency, (**b**) Schematic of experimental setup and measurement principle.

Figure 8a shows the photo of the experimental setup for measuring the fiber coupling efficiency, and Fig. 8b illustrates experimental setup and measurement principle in the form of a schematic sketch. The output power P_0_ of the light-emitting chip can be directly measured using a light power meter. The cap with a coupling aspheric lens is placed at a corresponding position in the optical path as shown in Fig. 8a, and the lens cap is adjusted to be coaxial with the optical axis. Manipulate the optical five-dimensional regulating platform to ensure that the laser beam through the cap aspheric lens can be focused on the receiving end face to couple the light into the single-mode fiber. Measure the light output power *P*_f_ at the other end face of the fiber using an optical power meter, then P_*f*_/ P_0_ is the coupling efficiency of the aspheric cap lens.

Table 3 presents the results of 10 measurements, among which the maximum coupling efficiency can be achieved is 88.63%, the minimum coupling efficiency is 69.82%, and the average coupling efficiency is 79.39%. The experimental maximum coupling efficiency of 88.63% is a little less than the theoretical value of 89.8%, which verifies experimentally the correctness of the theoretical value and also demonstrates effectiveness of the method for designing a coupling aspheric lens described in this paper.

**Table 3:** Experimental measurement of coupling efficiency.

No	1	2	3	4	5	6	7	8	9	10
Po (μW)	8095	8171	7034	7886	8049	7475	8303	8141	7222	8237
P_*f*_ (μW)	6845.48	7242.05	5064.42	5536.04	6754.8	5219.40	6826.50	6834.46	5763.98	6489.96
P_*f*_/ P_0_(%)	84.56	88.63	72	70.20	83.92	69.82	82.22	83.95	79.81	78.79

For comparison, we randomly selected 10 competing products from an internationally renowned brand. The coupling efficiency test is peformed under the same conditions, and the average coupling efficiency of 72.9267% is obtained. Obviously this coupling efficiency is relatively low in comparison with one using an aspherical cap lens in this design, which shows the ACA has the capabilities of reaching at good solutions to a lens design problem. However, several points need to be clarified. First of all, there are significant differences in lens material being used. Different lens materials have different light absorption rates. Secondly, alignment difficulties during cap lens assembly also affect the coupling efficiency.

From a design perspective, the ACA program automatically performe an iterative design without starting from a pre-existing human-created design, which is an improvement compared with the traditional design method. However, it can be seen from “Constructing the solution space, namely, the ant's position vector and its search range” section that the ACA method requires a structure parameter ranges to be given in advance. If the ranges selected are inappropriate, good results will be not achieved.

### Comparison Between a cap aspheric lens and an aspherical stigmatic lens

Next, we present rigorous analytical results for an bi-aspheric singlet lens free of spherical aberration using the version of Eq. (5) in Ref.^21^, so as to compare the difference of two designs. Given the conditions, ${ }t_{a} = - 0.975\;{\text{mm}}$ $t_{b} = 4.1531\;{\text{mm}}$, ${\text{t}} = 1.1019\;{\text{mm}}$, ${\text{n}} = 1.78331$, the first surface is a 8th order aspheric surface, and its equation is represented by the characters in Ref. 21.

11 $$ z_{a} = \frac{{C r_{a }^{2} }}{{1 + \sqrt {1 - \left( {1 + a_{2} } \right)C^{2} r_{a }^{2} } }} + a_{4} r_{a }^{4} + a_{6} r_{a }^{6} + a_{8} r_{a }^{8} $$

where C = 1/2.527396, *a*_2_ = − 22.415752, *a*_4_ = − 0.03073, *a*_6_ = 0.015847, *a*_8_ = 0.007523, as shown in Table 2.

Table 4 shows a set of analytical solutions $$r_{b}$$ and $$z_{b} $$ obtained by a set of known $$z_{a}$$-values, and the values of $${\text{z}}_{b}^{*}$$ are given by substituting $$r_{b}$$ into our optimized equation $${\text{ z}}_{b}^{*} = \frac{{C{ }r_{{b{ }}}^{2} }}{{1 + \sqrt {1 - \left( {1 + a_{2} } \right)C^{2} r_{{b{ }}}^{2} } }} + a_{4} { }r_{{b{ }}}^{4} + a_{6} { }r_{{b{ }}}^{6} + a_{8} { }r_{{b{ }}}^{8}$$, here,$${\text{C}},a_{2} ,{ }a_{4} { },a_{6} ,a_{8} { }$$ are shown in the third row of Table 2.

**Table 4:** The comparision between analytical and optimal solutions to design of singlet lenses free of spherical aberration.

rb	− 1.0360	− 0.9967	− 0.9552	− 0.9115	− 0.8657	− 0.8176	− 0.7672	− 0.7672	− 0.6598	− 0.6026
zb	0.5988	0.6349	0.6721	0.7100	0.7482	0.7864	0.8241	0.8609	0.8966	0.9307
zb*	0.5976	0.6344	0.6719	0.7100	0.7483	0.7864	0.8241	0.8609	0.8966	0.9307
rb	− 0.5431	− 0.4814	− 0.4175	− 0.3516	− 0.2837	− 0.2143	− 0.1436	− 0.0720	0	0.0720
zb	0.9629	0.9927	1.0198	1.0437	1.0640	1.0803	1.0922	1.0995	1.1019	1.0995
zb*	0.9628	0.9927	1.0198	1.0437	1.0640	1.0803	1.0922	1.0995	1.1019	1.0995
rb	0.1436	0.2143	0.2837	0.3516	0.4175	0.4814	0.5431	0.6026	0.6598	0.7146
zb	1.0922	1.0803	1.0640	1.0437	1.0198	0.9927	0.9629	0.9307	0.8966	0.8609
zb*	1.0922	1.0803	1.0640	1.0437	1.0198	0.9927	0.9628	0.9307	0.8966	0.8609
rb	0.7672	0.8176	0.8657	0.9115	0.9552	0.9967	1.0360			
zb	0.8241	0.7864	0.7482	0.7100	0.6721	0.6349	0.5988			
zb*	0.8241	0.7864	0.7483	0.7100	0.6719	0.6344	0.5976			

There are 37 groups of $z_{b}$ and ${\text{z}}_{b}^{*}$ in Table 4, namely, we trace 37 rays. Among them, there was a slight difference between $z_{b}$ and ${\text{z}}_{b}^{*}$ in 10 groups, and the rest is exactly the same. The above facts show that the optimization results are almost nearly perfect. We also noticed that the positions where $z_{b}$ are different from ${\text{z}}_{b}^{*}$ are on the edge of the lens. Authors think, the lens surface optimization in this paper is for the use of surface source only. However, an aspherical stigmatic lens design is for the axial point object. Due to tiny surface light sources, the difference between $z_{b}$ and ${\text{z}}_{b}^{*}$ is also very small.

We can fit a even aspheric polynomials equation in accordance with these data points of $r_{b}$ and $z_{b}$. For example, if, (1) even aspheric polynomials is ${\text{z}}_{b}^{*} = \frac{{C{ }r_{{b{ }}}^{2} }}{{1 + \sqrt {1 - \left( {1 + a_{2} } \right)C^{2} r_{{b{ }}}^{2} } }} + a_{4} { }r_{{b{ }}}^{4} + a_{6} { }r_{{b{ }}}^{6}$, then C = − 0.93711, a2 = − 1.19736, a4 = − 0.0327027, a6 = 0.0125095. (2) even aspheric polynomials is ${\text{z}}_{b}^{*} = \frac{{C{ }r_{{b{ }}}^{2} }}{{1 + \sqrt {1 - \left( {1 + a_{2} } \right)C^{2} r_{{b{ }}}^{2} } }} + a_{4} { }r_{{b{ }}}^{4} + a_{6} { }r_{{b{ }}}^{6} + a_{8} { }r_{{b{ }}}^{8}$, C = − 0.94228, a2 = − 1.47738, a4 = − 0.04612, a6 = − 0.00840588, a8 = 0.014156, etc.

## Conclusion

In this paper, we detailedly described the design process of a cap aspheric lens using ant colony optimization algorithm. A set of regular arrangements of variable parameters defining an aspheric lens is used as the position vector of ants. The coupling efficiency merit function for laser to single mode fiber is defined and calculated based on a ray-tracing technique, and the indirect functional relation between the merit function and the aspheric parameters is established. Taking the merit function as the pheromone of the ant colony algorithm, and the position vector with the global-best pheromone value is the best aspheric parameter combination.

Cap aspheric lens designed by self-complied MATLAB codes of ant colony algorithm, has the strong capability of coupling the emergent light of LD with a light-emitting area of 3 µm × 2 µm into a single-mode optical fiber. The divergence angle of emergent beam are about $\pm 35^\circ$ in the Y direction and $\pm 23.58^\circ$ in the Z direction, and a distance between a light-emitting surface and the receiving end of the optical fiber is 6.23 mm. The theoretical maximum coupling efficiency is 89.8%, and the experimental maximum coupling efficiency can reach up to 88.63%. The theoretical results are consistent with those from the experiment, indicating the correctness of the proposed design method. The coupling efficiency nearly 90% shows that the ACO-based algorithm has a noticeable performance at designing an cap aspheric lens.
